# Hyponatremia in babies: a 11-year single-center study

**DOI:** 10.3389/fped.2024.1338404

**Published:** 2024-06-06

**Authors:** Xu Liu, Yanshu Xie, Jing Tang, Jingzi Zhong, Dan Lan

**Affiliations:** Department of Pediatrics, First Affiliated Hospital of Guangxi Medical University, Nanning, China

**Keywords:** hyponatremia, aldosterone, pseudo-hypoaldosteronism, mineralocorticoid receptor, babies

## Abstract

**Introduction:**

Hyponatremia is one of the most prevalent water-electrolyte disturbances encountered in clinical practice in pediatrics and can arise from various conditions. However, there are limited reports on hyponatremia in hospitalized infants. The objective of this study was to provide an overview of the incidence, etiologies, and clinical characteristics of hyponatremia in hospitalized babies (from birth to 3 years old) at a tertiary hospital.

**Method:**

Computer records of all hospitalized babies (from birth to 3 years old) with hyponatremia were extracted from the First Affiliated Hospital of Guangxi Medical University's clinical databases.

**Results:**

801 patients from 39,019 hospital admissions were found to have hyponatremia and the overall prevalence of this condition was 2.05% in babies. Patients with hyponatremia due to aldosterone signaling abnormalities, neurological disorders, and liver diseases exhibited more severe outcomes than those with other etiologies.

**Conclusions:**

Various conditions can result in hyponatremia in hospitalized babies. Aldosterone signaling abnormalities were not that uncommon and it could lead to severe hyponatremia in babies.

## Introduction

A balance of water and electrolytes is essential for maintaining the normal biological processes in the human body. Sodium, the major electrolyte in extracellular fluid, plays a crucial role in stabilizing intracellular and extracellular volumes, as well as regulating acid-base balance (1, 2). Water balance and sodium homeostasis are closely interconnected. Thirst and the levels of antidiuretic hormone (ADH), which is secreted by the pituitary gland, are the principal mechanisms for regulating water metabolism. However, the concentration of sodium is mainly controlled by aldosterone. An excess of ADH can occur as a result of either hypovolemia or an inappropriate secondary secretion due to neurological disorders or pulmonary diseases, and these can increase the relative excess of water in the total body fluid, leading to hyponatremia. Aldosterone, the major mineralocorticoid hormone in humans, is responsible for the re-absorption of sodium as well as the excretion of potassium via the distal nephrons (3). Aldosterone signaling defects including defects in its biosynthesis [e.g., congenital adrenal hyperplasia (CAH), primary adrenal insufficiency (PAI) and isolated hypoaldosteronism] or aldosterone functional resistance (pseudo-hypoaldosteronism, PHA) may result in hyponatremia, especially in in babies (4, 5).

Hyponatremia is the most common electrolyte imbalance encountered in clinical practice, accounting for up to 20% of emergency hospital admissions, and it is a biochemical manifestation of various diseases (6, 7). In certain disorders, hyponatremia has been associated with disease severity, including increased mortality and hospital stay duration (8–10). Both acute hyponatremia (occurring within <48 h) and severe hyponatremia (serum sodium concentration <125 mmol/L) are considered as medical emergencies, since they can lead to serious neurological complications such as brain edema. Moreover, even mild hyponatremia has been linked to increased morbidity and mortality (11). Compared to adults, the occurrence of hyponatremia in infants is considered more dangerous due to their higher water and electrolyte requirements, as well as their weaker abilities to reabsorb sodium and water in urine. However, there are limited reports on hyponatremia in infants and young children.

The objective of this study was to assess all hospitalized babies (aged 0–36 months) in a tertiary hospital who presented with hyponatremia upon admission, in order to provide an overview of the incidence, etiologies, and clinical characteristics of this condition in this specific age group.

## Materials and methods

### Patients

A retrospective study was conducted in the First Affiliated Hospital of Guangxi Medical University, which is a tertiary medical center. Medical files of all hospitalized babies aged 0–36 months, who had at least once electrolyte concentration measurement from January 2012 to January 2023, were screened. All the patients with serum sodium concentration <135 mmol/L were included in our study. For those with several values, the lowest value was used for this study.

The exclusion criteria were patients with the following: hemolysis of blood specimen, known hyperglycemia or hyperlipoproteinemia and the impossibility of obtaining essential data from the patient's medical records.

### Data collection

All data regarding the patients' clinical and biological characteristics were collected from their medical records in order to identify the etiology of hyponatremia. The following information was gathered: demographics (age at admission, age at onset of hyponatremia, and sex), personal and previous medical histories (such as incidence of preterm birth, intestinal surgery, congenital malformation, or other disorders), family medical history (e.g., consanguinity and childhood deaths), in-hospital details (e.g., chief complaints and duration of hospitalization), clinical events preceding hyponatremia (e.g., gastrointestinal loss, sepsis, heart failure, urinary tract disorders, and neurological disease), ongoing therapy (e.g., diuretics, intravenous fluid administration, and chemotherapy drugs), biochemical tests (e.g., electrolytes, glucose, routine blood measurements, liver and renal functions), and discharge status. Additionally, imaging test results, hormonal characteristics, and genetic mutation analysis for patients suspected of having endocrine disorders were also included.

### Definitions

Hyponatremia was defined as a serum sodium concentration <135 mmol/L, with mild hyponatremia defined as levels between 130 and 135 mmol/L. Moderate hyponatremia was categorized as a serum sodium concentration between 125 and 129 mmol/L, and levels below 125 mmol/L were classified as severe hyponatremia (7, 12).

Aldosterone signaling abnormalities, including impairment in its biosynthesis (e.g., CAH, PAI and isolated hypoaldosteronism) as well as functional resistance (e.g., pseudo-hypoaldosteronism). Diagnosis of a disease was based on its practical guidelines (13, 14). For rare cases with no guidelines, this mainly depended on the patient's clinical symptoms, biochemical parameters and molecular genetic tests.

Patients with urinary tract infections (UTIs) such as obstructive uropathy (OUP), urinary tract malformations (UTM) and intestinal tract disease (e.g., ileostomy and jejunal membrane-associated complications) who present with hyponatremia, metabolic acidosis as well as hyperkalemia would usually be suspected of having transient pseudo-hypoaldosteronism (PHA).This would subsequently be confirmed by elevated serum levels of aldosterone.

### Statistical analysis

All statistical analyses were performed with SPSS 26.0 software (IBM Corp., Armonk, NY, USA). Normally distributed data with were expressed as means ± standard deviations and were compared using either a two-independent sample *t*-test or one-way analysis of variance. Data not normally distribution were expressed as medians (with the minimum and maximum ranges) and were compared by using either the Whitney *U* or Kruskal–Wallis *H* tests. *P*-values <0.05 were considered to be statistically significant.

## Results

A total of 39,019 patients aged under 36 months were admitted to our hospital from January 2012 to January 2023. Among them, 1,890 patients were found to have electrolyte disturbances, with 829 of these experiencing an episode of hyponatremia. However, 28 patients were later excluded from the study: 27 individuals had highly hemolyzed blood samples, and subsequent biochemical tests conducted in the following days showed normal serum sodium levels. Additionally, one infant was diagnosed with familial hyperlipidemia due to a genetic disorder. Ultimately, 801 patients who presented with an episode of hyponatremia were included in this study.

### Demographic characteristics and patient outcomes

**Figure 1:**
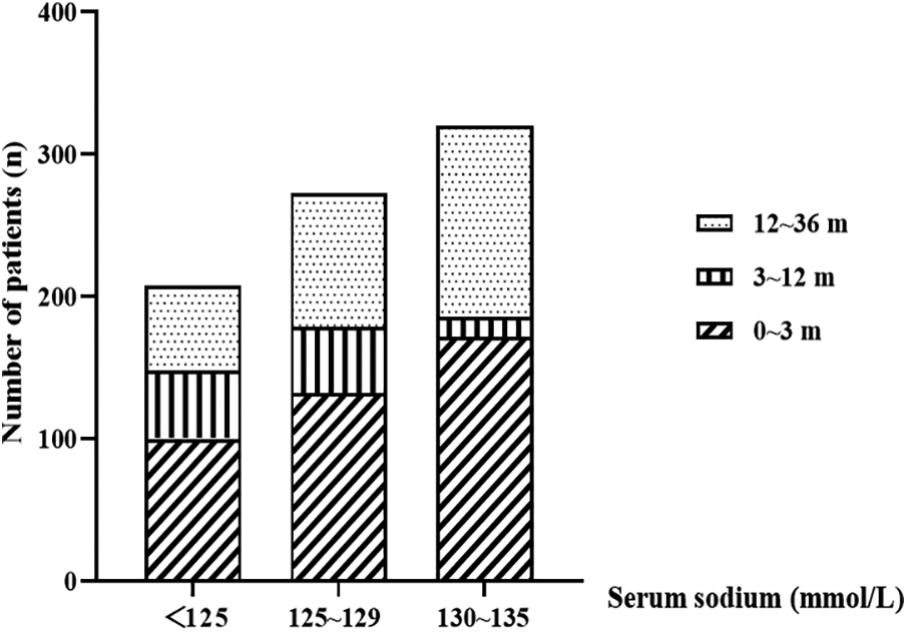
The distribution of serum sodium concentrations in patients based on their age. m: month.

Among the 801 patients enrolled in the study (515 males and 286 females), the median age at onset of hyponatremia was 2.57 months (range: 0.00–36.00 months). Of these patients, 404 (50.4%), 109 (13.6%), and 288 (36.0%) fell into the age groups of 0–3, 3–12, and 12–36 months, respectively (Table 1). Within 48 h of hospitalization, 215 patients (26.8%) experienced an episode of hyponatremia. The serum sodium concentrations ranged from 94.1 to 134.9 mmol/L, with a median value of 128.8 mmol/L. Among the patients, 320 (39.9%), 273 (34.1%), and 208 (26.0%) were diagnosed with mild, moderate, and severe hyponatremia, respectively. Infants aged 0–3 months constituted the largest proportion in all hyponatremia groups (Figure 1). Additionally, 56 cases (7.0%) presented with hyponatremia accompanied by convulsions, and 118 patients (14.7%) had poor prognoses, including death or discharge for palliative care. Furthermore, two patients had prognoses closely associated with severe hyponatremia.

**Table 1:** The characteristics of the babies aged 0–3 years who presented with an episode of hyponatremia.

Patients (*n*, %)	
Total	801 (100.0)
Male	515 (64.3)
Preterm birth	179 (22.3)
Patients aged 0–3 m	404 (50.4)
Patients aged 3–12 m	109 (13.6)
Patients aged 12–36 m	288 (36.0)
Chief complaints (*n*, %)	
Vomiting	229 (28.6)
Fever	306 (38.2)
Diarrhea	154 (19.2)
Dehydration	86 (10.7)
Abdomen distention	82 (10.2)
Failure to thrive	77 (9.6)
Oliguria	51 (6.4)
Consciousness	8 (1.0)
Dyspnea	121 (15.1)
Edema	41 (5.1)
Hyponatremia occurred 48 h after hospitalization	215 (26.8)
Poor prognosis	118 (14.7)
Range^a^	
Age at hyponatremia onset (months)	2.6 (0.0–36.0)
Serum sodium concentration (mmol/L)	128.8 (94.1–134.9)
Length of hospital stay (days)	11.0 (0.0–98.0)

^a^ Median (minimum–maximum). M, male; F, female; d, days; m, months.

### Etiologies of hyponatremia

Hyponatremia could have resulted from a wide spectrum of conditions in infants and young children including aldosterone signaling abnormalities, sepsis and neurological disorders as well as diseases of the respiratory, hematological, hepatic, urinary, gastrointestinal and cardiovascular systems in our study (Table 2). Multiple systems disorders were also included. In addition, medical interventions including those of iatrogenic origin (e.g., diuretics, chemotherapeutic agents and hypotonic fluid infusion) could also lead to hyponatremia. The commonest three etiologies of hyponatremia in this study were gastrointestinal system disease, sepsis and respiratory system disease which occurred in 284 (35.5%), 93 (11.6%) and 87 patients (10.9%), respectively. Among patients with hyponatremia younger than 3 months of age, the top three causes were gastrointestinal diseases (180/404, 44.6%), respiratory diseases (71/404, 17.6%), and aldosterone signaling abnormalities (39/404, 9.7%). Among patients with severe hyponatremia, gastrointestinal diseases (57/208, 27.4%) and aldosterone signaling abnormalities (36/208, 17.3%) accounted for the largest proportion, respectively.

**Table 2:** Characteristics of hospitalized babies presented with hyponatremia in different etiologies.

Diseases	Proportion of patients in this study, *n* (%)	Mild hyponatremia *n* (%)	Moderate hyponatremia *n* (%)	Severe hyponatremia *n* (%)	Age 0–3 m *n* (%)	Age 3–12 m *n* (%)	Age 12–36 m *n* (%)
Gastrointestinal system disease	284 (35.5)	119 (37.2)	108 (39.6)	57 (27.4)	180 (44.6)	35 (32.1)	69 (24.0)
Sepsis	93 (11.6)	35 (10.9)	34 (12.4)	24 (11.5)	23 (5.7)	17 (15.6)	53 (18.4)
Respiratory system disease	87 (10.9)	39 (12.2)	31 (11.3)	17 (8.2)	71 (17.6)	7 (6.4)	9 (3.1)
Cardiovascular disease	60 (7.5)	24 (7.5)	26 (9.5)	10 (4.8)	28 (6.9)	11 (10.1)	21 (7.3)
Urinary system disease	59 (7.4)	16 (5.0)	25 (9.2)	18 (8.7)	8 (2.0)	8 (7.3)	43 (14.9)
Multi-system disease	54 (6.7)	33 (10.3)	10 (3.7)	11 (5.3)	22 (5.4)	8 (7.3)	24 (8.3)
Neurological disorder	53 (6.6)	13 (4.1)	16 (5.9)	24 (11.5)	17 (4.2)	7 (6.4)	29 (10.1)
Aldosterone signaling abnormalities	52 (6.5)	4 (1.2)	12 (4.4)	36 (17.3)	39 (9.7)	9 (8.3)	4 (1.4)
Iatrogenic origin	36 (4.5)	28 (8.8)	4 (1.5)	4 (1.9)	9 (2.2)	1 (0.9)	26 (9.0)
Liver disease	14 (1.7)	3 (0.9)	5 (1.8)	6 (2.9)	7 (1.7)	6 (5.5)	1 (0.3)
Hematological system disease	9 (1.1)	6 (1.9)	2 (0.7)	1 (0.5)	0 (0.0)	0 (0.0)	9 (3.1)
Total numbers	801 (100.0)	320 (100.0)	273 (100.0)	208 (100.0)	404 (100.0)	109 (100.0)	288 (100.0)

Mild hyponatremia: serum sodium concentration range 130–135 mmol/L; Moderate hyponatremia: serum sodium concentration range 125–129 mmol/L; Severe hyponatremia: serum sodium concentration <125 mmol/L. Defects in aldosterone including defects in biosynthesis and function; m: months.

Aldosterone signaling abnormalities including biosynthesis defects and functional resistance were identified in 52 (6.5%) patients, of which CAH due to 21 hydroxylase deficiency and PAI occurred in 32 and 4 patients, respectively. Isolated hypoaldosteronism due to novel compound heterozygous mutations of the CYP11B2 gene and PHA type 1 due to a novel heterozygous mutation of NR3C2 gene each occurred in one patient. 13 patients were considered as transient PHA (in 6 infants this was secondary to urinary tract disorders and in 7 patients this due to intestinal tract disorders). Additionally, one young child presented with difficulties in walking as well as a high creatine kinase level and low serum cortisol and hyponatremia was finally confirmed with Xp21 contiguous gene deletion syndrome.

**Figure 2:**
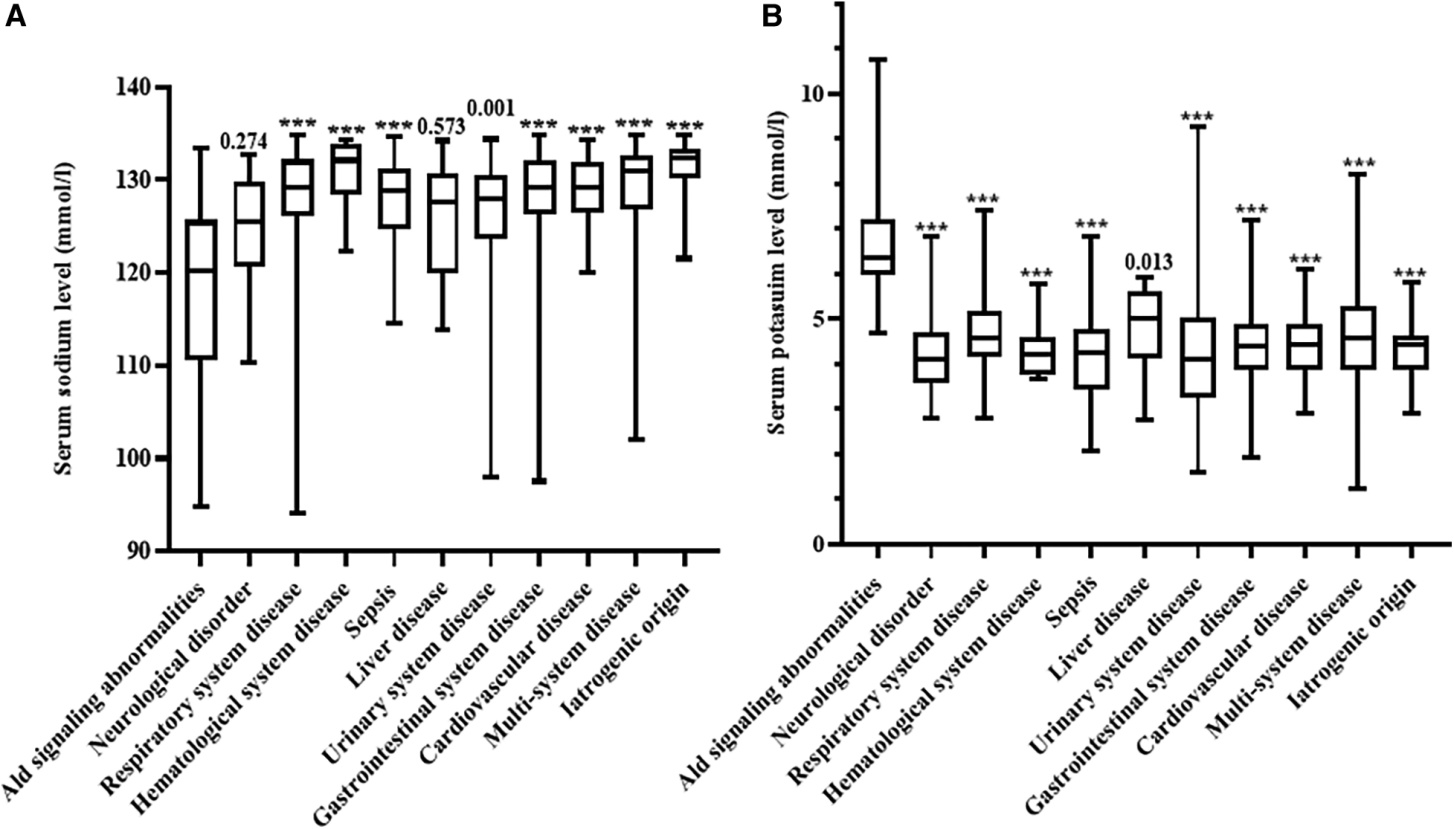
Comparison of serum sodium and potassium concentrations in the different disorders noted in our patients. (**A**) Comparison of serum sodium in the different disorders noted in our patients; (**B**) comparison of serum potassium concentrations in the different disorders noted in our patients. Ald, aldosterone; ns, not significant. ***P < 0.001.

Serum sodium and potassium concentrations were significantly different between the different etiologies of hyponatremia (Figure 2). Patients with aldosterone signaling abnormalities had more severe hyponatremia than those with other diseases except for neurological disorders and liver diseases. Serum potassium concentrations were also significantly higher in patients due to aldosterone signaling abnormalities than in those with all other etiologies (*P* < 0.05).

## Discussion

Hyponatremia was found to be a relatively common disorder in hospitalized patients. The incidence of hyponatremia in hospitalized babies aged 0–36 months was 2.05% (801/39,019 patients). Wattad et al. (15) conducted a study on hospitalized children over a 12-month period and the results showed that the overall frequency of hyponatremia was 1.38%. Moreover, Storey et al. (16) found that neonatal hyponatremia had a prevalence of 4.3%. Our study is the first study to describe the incidence, etiologies and clinical characteristics of hyponatremia in babies. Hyponatremia was found to be highly heterogeneous and based on causes with numerous different conditions (15–17). In our study, the top three causes of hyponatremia in babies were gastrointestinal system disease, sepsis and respiratory system disease. This was probably due to the incompletely development of the digestive and gastrointestinal systems in infants and young children. Recurrent vomiting and diarrhea due to gastrointestinal system disease can result in hyponatremia and these clinical symptoms were commonly encountered in our cohort. Sepsis can also reduce the circulating blood volume and it may cause baroreceptor activation and ADH release, leading to hyponatremia (6, 18).

Our findings confirmed the findings of some previous studies (19, 20). Hyponatremia usually results from respiratory system diseases including respiratory tract infections, pneumonia and bronchiolitis in children. Additionally, hyponatremia due to an iatrogenic origin is not unusual. Diuretics and hypotonic fluid infusion were the main iatrogenic causes of hyponatremia. However, in our cohort, hyponatremia also occurred in 10 cases with acute lymphoblastic leukemia (ALL) following vincristine therapy. It is reported that vincristine may have some neurotoxic effects which are towards the hypothalamus and pituitary glands, resulting in hyponatremia (21). Similar results were found in several other ALL pediatric cohorts (22, 23). This indicates that intensive monitoring of the serum sodium levels is an essential requirement for children on vincristine therapy and diuretic treatments. Moreover, the use of hypotonic fluid infusion should be carefully assessed in order to prevent iatrogenic hyponatremia.

Aldosterone biosynthesis defects or aldosterone resistance was also relatively common in our cohort. Besides the salt-losing associated with CAH and PAI, we also identified some rare etiologies including isolated hypoaldosteronism, PHA type 1, transient PHA. PHA type 1 and transient PHA are described as mineralocorticoid resistance. These two diseases have similar clinical features including hyponatremia, hyperkalemia acidosis and elevated serum aldosterone during infancy. However, PHA type 1 is a genetic disorder and transient PHA is secondary to disorders of urinary and intestinal tracts and the resultant hyponatremia that arise from these conditions, can be corrected with improvement of the primary disease (5, 24).

To date, more than 130 cases of transient PHA have been reported and these are mainly secondary to urinary tract infections (UTIs) and UTMs during infancy (25–27). However, up to now, only a few cases secondary to UTIs and/or UTMs have been documented in China (28, 29). In this study, we identified six infants in UTIs and/or UTMs presenting with transient PHA and the oldest patient was 6.43 months. The incidence of transient PHA is still unclear. A survey of the Irish population found that transient PHA occurs at a rate of 1 per 13,200 total live births per year (30). However, the authors only included the patients who had disorders of the urinary tract (31–37). Intestinal tract disorders can also result in transient PHA, and we reviewed these cases in Supplementary Table S1. When compared with UTIs and UTMs, transient PHA secondary to intestinal tract disorders can occur at any age, especially in patients with ileostomy. The mechanism of transient PHA is still unclear. Bacterial endotoxins and several cytokines including transforming factor-β1, IL-1 and TNF-α may act on the relatively immature young kidneys and this may be the underlying mechanism in patients with transient PHA secondary to UTIs and UTMs (26, 38). Whereas, the hypothesis in PHA secondary to intestinal is probably very different. A high over output of electrolytes probably results in chronic sodium depletion and hypovolemia leading to severe secondary hyperaldosteronism. This would eventually trigger PHA and cause the down-regulation of mineralocorticoid receptors which may result in the development of secondary PHA due to intestinal tract disorders (31, 34).

More experiments are needed to corroborate these hypotheses. In addition, the four of the six infants with PHA secondary to UTIs in our cohort, showed no fever. This reminds us that pediatricians seeing any infant presenting with unexplained hyponatremia should perform urine test cultures and renal ultrasound.

**Figure 3:**
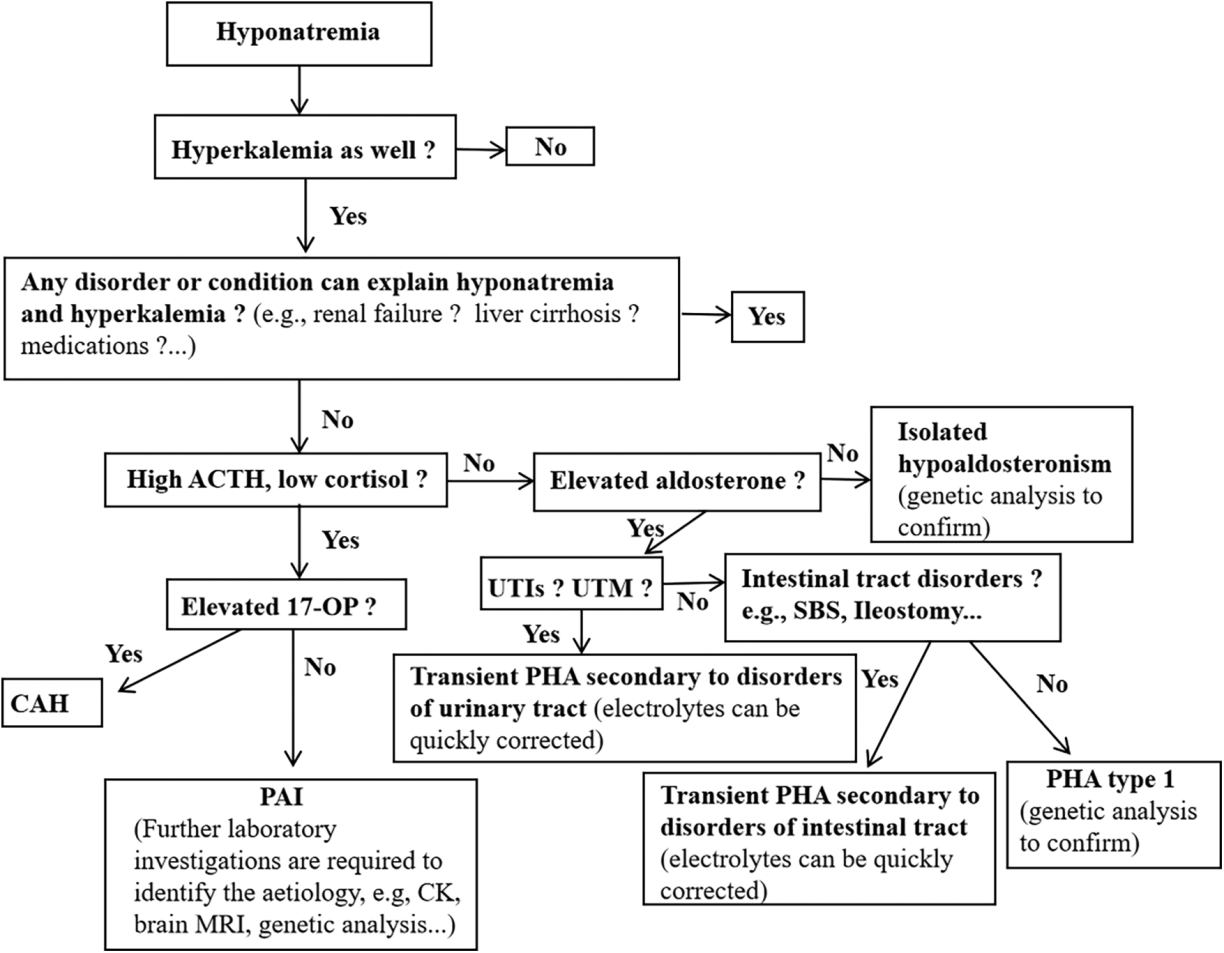
Proposed a brief diagnostic flow chart for inants and young children presenting with hyponatremia who is suspected of aldosterone signaling abnormalities. Ald, aldosterone; CAH, congenital adrenal hyperplasia; PHA, pseudohypoaldosteronism; PAI, primary adrenal insufficiency; 17-OHP, 17-hydroxyprogesterone; CK, creatine kinase; UTIs, urinary tract infections; UTM, urinary tract malformations.

Our study found that the serum sodium concentrations differed considerably between the different diseases. Hyponatremia due to aldosterone signaling abnormalities, neurological disorders and liver diseases were generally more severe conditions when compared to the other diseases (Figure 2A). In addition, serum potassium concentrations were also significantly different between the different causes of hyponatremia (Figure 2B). These results remind pediatricians should consider aldosterone signaling abnormalities in babies presented with severe hyponatremia and hyperkalemia. Furthermore, we further propose a specific diagnostic flow chart to facilitate the accurate diagnosis of babies who present with hyponatremia where aldosterone signaling abnormalities are suspected (Figure 3).

## Conclusions

This study described the prevalence, etiologies and clinical characteristics of hyponatremia in hospitalized infants and young children. The findings showed that hyponatremia is a relatively common electrolyte disorder in hospitalized babies and it can result from a wide spectrum of conditions. The top three causes of hyponatremia in babies were found to be gastrointestinal system disease, sepsis and diseases of the respiratory system. Additionally, hyponatremia due to aldosterone signaling abnormalities was not unusual. We further proposed a specific flow chart to help pediatricians to investigate the aldosterone pathway abnormalities in babies with hyponatremia. This study had many limitations due to its retrospective nature. Larger sample sizes as well as prospective and long-term follow-up studies are required to fully understand the mechanisms and the outcomes of hyponatremia in young children.

## Data Availability

The datasets presented in this study can be found in online repositories. The names of the repository/repositories and accession number(s) can be found in the article/Supplementary Material.
