# Supplementary material for: Hyponatremia in babies: a 11-year single-center study
Source: Front Pediatr. 2024 Jun 6;12:1338404. doi: 10.3389/fped.2024.1338404 (PMC11187253; doi:10.3389/fped.2024.1338404)
Supplement: Supplementary file 1 [file Table1.doc]

**Supplementary Table. 1**: Review of the characteristics of PHA secondary to gastrointestinal tract losses

| Author  date | Age at diagnose | Sex | Serum Na  (mmol/l) | Serum K  (mmol/l) | Serum Cl  (mmol/l) | Renin or PRA | Serum Ald  (pg/ml) | Metabolic acidosis | Clinical symptoms | Foundation disease or conditions | Treatments |
| --- | --- | --- | --- | --- | --- | --- | --- | --- | --- | --- | --- |
| Nissen  2017 (33) | 50 d | M | 105 | 6.4 | 70 | H | 1170 | + | Vomiting | Congenital jejunal membrane | Rehydration,  Electrolyte replacement  HC, surgery |
| Vantyghem  1999 (34) | 54 y | F | 118 | 7.6 | 87 | H | >2000 | + | Fatigue  Severe nausea | Ileostomy Colectomy | Rehydration  Salt supplementation  FC, HC |
| Sugawara  1989 (35) | 82 y | M | 125 | 6.1 | 90 | H | 120000 | NA | None | Ileostomy Colectomy | Intravenous fluids |
| Nakasone  2021 (31) | 22 d | F | 124 | 6.4 | 94 | H | 4480 | _ | Oliguria | Ileostomy | Sodium  supplementation |
|  | 145 d | M | 133 | 9.6 | 99 | H | 9190 | + | Cardiac arrest | Ileostomy | Sodium  supplementation  with GI therapy |
|  | 96 d | M | 121 | 7.3 | 86 | H | 15000 | _ | None | Ileostomy | Sodium  supplementation  with GI therapy |
| Ou  2021 (32) | 30 d | M | 126 | 6.12 | NA | NA | >16300 | + | Dehydration  Weight loss | Ileostomy | IV fluids  Salvage operation |
|  | 62 d | F | 126 | 5.31 | NA | H | >16300 | + | None | Ileostomy | IV fluids  Salvage operation |
|  | 39 d | F | 122 | 6.85 | NA | H | >16300 | + | None | Ileostomy | IV fluids  Salvage operation |
|  | 89 d | M | 125 | 6.8 | NA | NA | 14710 | _ | Bradycardia  Weight loss | Jejunostomy | IV fluids  Salvage operation |
|  | 62 d | M | 120 | 3.4 | NA | NA | 3720 | _ | Dehydration  Weight loss | Ileostomy | IV fluids  Salvage operation |
|  | 38 d | M | 126 | 5.1 | NA | H | 3460 | _ | Dehydration  Oliguria | Ileostomy | IV fluids  Salvage operation |
|  | 62 d | M | 125 | 4.4 | NA | NA | 3830 | _ | Cardiac arrest | Ileostomy | IV fluids  Salvage operation |
| Niyazov  2017 (36) | 65 y | F | 132 | 5.0 | NA | H | 3355 | NA | Low appetite  Weight loss  Hypotension | Ileostomy | Ileostomy reversal |
| Alassaf  2015 (37) | 1 m | F | 126 | 7.0 | NA | Normal | 6370 | + | Dehydration | Ileostomy | IV fluids, HC  Sodium polystyrene Closure of ileostomy |

PHA: pseudohypoaldosteronism; F: female; M: male; d: days; m: months; y: years; Na: sodium; K: potassium; Cl: chloride; Ald: aldosterone; H: high; HC: hydrocortisone; FC: fluorohydrocortisone; GI therapy: glucose-insulin therapy; NA: not available.
